# Laboratory Tests and Analyses of the Level of Vibration Suppression of Prototype under Ballast Mats (UBM) in the Ballasted Track Systems

**DOI:** 10.3390/ma14020313

**Published:** 2021-01-09

**Authors:** Cezary Kraśkiewicz, Artur Zbiciak, Kacper Wasilewski, Anna Al Sabouni-Zawadzka

**Affiliations:** 1 Institute of Roads and Bridges, Faculty of Civil Engineering, Warsaw University of Technology, Al. Armii Ludowej 16, 00-637 Warsaw, Poland; a.zbiciak@il.pw.edu.pl; 2 Institute of Building Engineering, Faculty of Civil Engineering, Warsaw University of Technology, Al. Armii Ludowej 16, 00-637 Warsaw, Poland; k.wasilewski@il.pw.edu.pl (K.W.); a.sabouni@il.pw.edu.pl (A.A.S.-Z.)

**Keywords:** under ballast mats, vibration isolation, rheological model, testing procedures

## Abstract

The present paper is aimed at the analysis of under ballast mats (UBM) which are used in ballasted track structures as vibration isolators and to protect the ballast layer against fast degradation. The mats were tested in the laboratory and afterwards a novel 4-DoF mechanical model of the track structure with UBM was developed. The novelty of this study consists in the comparison of two testing procedures: a procedure based on the popular German standard DIN 45673-5 and a new European standard EN 17282, released in October 2020. Major discrepancies were demonstrated in the determined values of the static and dynamic characteristics using both approaches—especially in reference to the mats with higher stiffness.

## 1. Introduction

The first applications of under ballast mats (UBM) date back to the 1960s in France. In the following decades various tests of UBM have been performed: starting from the 1970s in Germany, the 1980s in Switzerland and then, from the 1990s in Italy and Belgium [1]. Two types of tests, which are correlated with two main functions of the resilient mats, may be distinguished. Their first function is to reduce the level of vibration caused by the rolling stock, the second—to protect the ballast layer against fast degradation caused by the abrasion and breakage of the ballast grains.

In reference to the vibration isolation function of UBM, the works of Wettschureck [2,3,4] should be mentioned. He analyzed the effectiveness of the vibration isolation using a track structure model with one degree of freedom (1-DoF). The discrepancies between the predicted level of the vibration suppression determined using a 1-DoF analytical model of the track structure with UBM and the results of measurements performed on the real track in the high range of frequencies (over 100 Hz) are discussed in [5]—as a conclusion it is recommended to use models with more degrees of freedom. The work [5] has encouraged the authors of this paper to develop and present a more advanced 4-DoF model of the track system, which includes resilient elements.

Examples of practical protection of buildings against negative influence of vibration coming from the railway line in a tunnel are shown in [6,7]. Moreover, these works contain results of laboratory tests on the durability of static and dynamic parameters after seventeen and thirty years of operation. A small variability of isolation effectiveness of UBM during their long-term use was observed, which was additionally confirmed in the field tests.

Numerical analyses of the reduction of vibration level by using UBM are presented in [8], where various variants of the mats stiffness and their location in the track structure (under the ballast or under the protection layer in the substructure) were considered. It was proved that the reduction of the mat stiffness causes an increase of the vibration isolation effectiveness and it moves the region of effective vibration isolation in the direction of smaller frequencies. Moreover, it was highlighted that the deflections of the track structure need to be considered while determining the minimum stiffness of the mat. Xin et al. [9] analyzed rubber mats used in transition zones between two slab tracks. Rubber-based UBM were applied to solve the transition problem by gradually changing the stiffness of the system.

Applications of UBM as the elements that are aimed at reducing the breakage of the ballast grains caused by dynamic effects are presented in [10], where results of laboratory impulse tests and numerical simulations are described. The authors of this work confirmed the purposefulness of the application of UBM, particularly with a rigid subgrade of the ballast layer—for example a steel or concrete ballast trough which is used in track structures that are located on bridges or in tunnels. Kumar et al. [11] proposed a discrete element method (DEM) to analyze the railway ballast behavior under cyclic loading in the systems with under sleeper pads (USP) and UBM. Qu et al. [12] focused on the analysis of the vibration mitigation properties of ballasted ladder tracks with elastic elements. A finite element–infinite element (FE-IFE) model is proposed to investigate the behavior of vibration isolators.

Diversification (up to 30%) of the values of bedding moduli of UBM determined in the laboratory tests, depending on the ballast plate type and the support conditions, is discussed in [13]. This work has been used as a basis for the comparative study of static and dynamic parameters of UBM tested using two types of ballast plates, which is presented in the present paper.

There are many works that are aimed at finding solutions to enhance the vibration isolation effectiveness of UBM and other resilient elements used in track structures. In [14] mechanical properties of rubber-based UBM used in high-speed railway systems were investigated. Results of various static, dynamic and fatigue tests were presented and performance of UBM as the isolation layer was analyzed. The papers [15,16,17] focus on the analysis of rail pads, under sleeper pads (USP) and under ballast mats (UBM) manufactured from deconstructed end-of-life tires. The proposed resilient elements were applied in order to reduce global stiffness of the ballasted track structure and to reduce the vibration level. In [18] static and dynamic characteristics of the track with rubber-based UBM produced from end-of-life tires were determined. A positive effect of the tested vibration isolators on eigenfrequencies of the track structure was demonstrated. It was also stated and justified that the deflections of the track structure should be considered while determining material characteristics of the mats. The authors in [19] tested the characteristics of isolation layers in unit-plate ballastless track systems. They concluded that these layers influence vibration modes and transmission properties of the ballastless tracks and as a result, the vibrations become stable. Another interesting group of papers [20,21,22] is dedicated to various reinforcing systems used in track structures, such as geotextiles and geomembranes applied as separators between the ballast and the subgrade. They are used as filter layers which improve track stability and reduce the maintenance costs.

The present paper focuses on the laboratory tests aimed at identifying static and dynamic characteristics of prototype UBM. The novelty of this study consists in the comparison of two testing procedures: a procedure based on the popular German standard DIN 45673-5 [23] and a new European standard EN 17282 [24], released in October 2020. Major discrepancies in the values of static and dynamic characteristics determined using both approaches were demonstrated—especially in reference to the mats with higher stiffness (a medium and stiff type). The values of static and dynamic parameters were used to develop a viscoelastic rheological model of the vibration isolator UBM, which includes fractional elements. The proposed original model with four degrees of freedom (4-DoF) is used by the authors to prove that the adopted testing procedure has a significant influence on the results of analyses of the level of vibration suppression, expressed by the insertion loss factor.

## 2. Testing Procedures and Requirements for UBM

### 2.1. Standards and Testing Procedures of UBM

**Figure 1:**
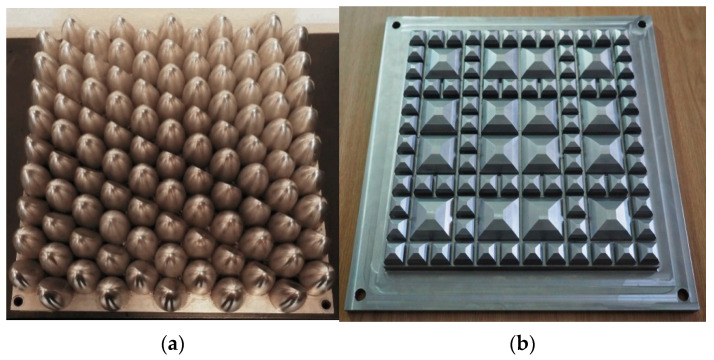
Ballast plates used in UBM tests: (**a**) formed steel plate used in fatigue tests according to UNI 11059 [27]; (**b**) geometric ballast plate (GBP) according to EN 17282 [24].

Up to now, the majority of manufacturers of under ballast mats have used testing procedures described in the German standard DIN 45673-5 [23] or Technical delivery conditions specified by the German Infrastructure Manager BN/DBS 918 071-01 [25,26]. The German standard [23] specifies unified procedures that should be used in laboratory tests that are aimed at identifying parameters of UBM (for example static and dynamic bedding moduli), but it does not specify limiting values of these parameters. Technical delivery conditions [25,26], on the other hand, specify selected testing procedures and required values of selected mats parameters and thus, make it possible to classify UBM with respect to the function (ballast protection or vibration isolation). Among other approaches, the procedures included in Italian standards UNI 11059 [27] and UNI 10570 [28] have also been often used. It should be mentioned that according to German (DIN) and Italian (UNI) standards the tests should be performed using specimen with the dimensions of 300 mm × 300 mm and flat ballast plates and Technical delivery conditions (BN/DBS) require the specimen dimensions from 300 mm × 300 mm to 500 mm × 500 mm. The fatigue test of UBM described in UNI 11059 [27] is an exception—in this case the specimen of 300 mm × 300 mm should be tested using a formed steel plate shown in Figure 1a.

The new standard released in 2020—EN 17282 [24]—is based on the tests performed on the samples 300 mm × 300 mm with the use of a geometric ballast plate (GBP) (the same as used for USP tests according to PN-EN 16730 [29], as presented in [30]), shown in Figure 1b. Its irregular structure should simulate behavior of UBM in the ballasted track system.

Results of vibration isolators (USP and UBM) tests that are carried out in the same range of loads and frequencies (for dynamic tests), but with different ballast plates, differ significantly. This was proved in [31] for USP and for UBM—the differences between such tests are shown in the next section of this paper. Therefore, it is necessary to verify the products, which were previously tested in accordance to the German or Italian standards, by retesting them according to the new European standard EN 17282 [24] with the use of GBP.

The procedures described in the European standard are to a certain degree consistent with the ones included in the German standard. However, there are some discrepancies that should be discussed. Apart from the already mentioned aspect related to the type of a ballast plate, the new standard specifies more precisely how the fatigue tests should be performed. For the UBM fatigue tests with the use of a ballast trough, the European standard specifies the refinement level of the ballast and gives requirements for the material of the ballast (resistance to fragmentation and resistance to wear), which ensures greater repeatability of measurements. Additionally, according to EN 17282 [24] the tested UBM samples should consist of two identical parts connected together, in order to verify the durability of the connection. The most important modification, however, is the changed number of loading cycles in the fatigue tests in the ballast trough—according to DIN 45673-5 [23] it was 12.5 million cycles, and EN 17282 [24] requires only 2.5 million cycles.

### 2.2. Requirements for UBM

In this section the authors gathered requirements for the UBM that are valid in German, Austrian and Swiss railways.

#### 2.2.1. German Railways

The first document that specifies requirements for using UBM in German railways (DB, Deutsche Bahn)—Technical delivery conditions (TL, Technische Lieferbedingungen) TL 918 071 [32]—was released in 1988. In 2000 the document was integrated with German railway standards (BN, Bahn Norm) and released as BN 918 071-1 [25]. The first part of this document included UBM, applied to increase the elasticity between the ballast and its rigid subgrade and to protect the ballast layer by reducing stresses. The second part of this document BN 918 071-2 should concern UBM aimed at reducing the vibration level, however, to the best knowledge of the authors, this part has not been released. Afterwards, in 2006, another document was introduced and adopted as German Railway Standards (DBS, Deutsche Bahn-Standard) DBS 918 071-01 [26].

Regulations of DB [25,26] that specify requirements for static bedding moduli of UBM applied as protection of the ballast layer are given in Table 1. A static bedding modulus $C_{\mathrm{stat}}\;\left[N/{\mathrm{mm}}^{3}\right]$ is a ratio of the static load, applied to the sample with a certain cross-sectional area, to the sample deflection caused by this load. It characterizes deflection of the track system under a nonmoving rolling stock.

Regulations of DB [1,33,34] that specify requirements for static bedding moduli of UBM applied as vibration isolators are given in Table 2.

#### 2.2.2. Austrian Railways

Regulations of Austrian railways (ÖBB, Österreichische Bundesbahnen) Richtlinien B50-1 [35] specify requirements for static bedding moduli of UBM which are collected in Table 3.

#### 2.2.3. Swiss Railways

Regulations of the Swiss railway manager (SBB CFF FFS—Schweizerische Bundesbahnen; Chemins de fer Fédéraux Suisses; Ferrovie Federali Svizzere) [1,36] specify requirements for the UBM parameters, from which the most important ones (from the point of view of vibration isolation and deflection values) are gathered in Table 4.

## 3. Laboratory Tests of Static and Dynamic Characteristics of UBM according to DIN 45673-5 and EN 17282

The main aim of the performed laboratory tests was to compare the values of static and dynamic bedding moduli in low and high frequencies, using eighteen samples of prototype UBM coming from the same lot, carried out according to the procedures described in:

- The German standard DIN 45673-5 [23];
- The new European standard EN 17282 [24].

Based on the regulations of foreign railway infrastructure managers collected in Section 2, guidelines for laboratory identification tests performed according to [23] were formulated in Table 5.

**Figure 2:**
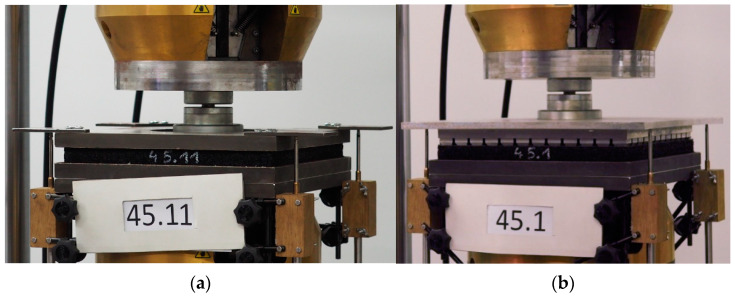
UBM tests: (**a**) with a flat ballast plate according to DIN 45673-5 [23]; (**b**) with a geometric ballast plate (GBP) according to EN 17282 [24]. The tested UBM specimen is a rubber-based mat no. 45, 20 mm thick.

The test stand consisted of a universal testing machine Instron 8802 (Figure 2; Instron, Norwood, MA, USA) with two steel plates: a bottom support plate 320 mm × 320 mm and a top ballast plate—flat (Figure 2a) or GBP (Figure 2b). During the tests displacements of four points were measured using a test kit consisting of four inductive displacement transducers WA-T (by HBM, Hottinger Baldwin Messtechnik GmbH, Darmstadt, Germany), a signal conditioning system Spider8 and a software Catman AP (version 3.4).

The configuration of the test stand for UBM tests according to DIN 45673-5 [23] is presented in Figure 2a. Moreover, in order to meet requirements specified in the new standard EN 17282 [24], a configuration of the test stand with GBP was prepared (Figure 2b).

The tested specimen of eighteen prototype UBM had dimensions of 300 mm × 300 mm and a varying thickness between 15 mm and 35 mm. The samples based on mineral wool were designated with numbers 37–39, the rubber-based (SBR—styrene-butadiene rubber) samples—42–56. The properties of tested samples are presented in Table 6. Samples based on mineral wool have the same density and thickness but they differ in production technology.

In the present paper the authors focused mainly on two rubber-based mats:

- UBM 042—thickness 25 mm, density 850 kg/m^3^, very stiff type, main function: the ballast protection;
- UBM 045—thickness 20 mm, density 700 kg/m^3^, medium type, main function: vibration isolation.

The variations of bedding moduli of UBM (Δ*C*), tested according to two different procedures, were classified with the use of four colors in Table 7.

Additionally, for two selected UBM samples (042 and 045) the static and dynamic (in low and high frequencies) bedding moduli, obtained using two procedures, were compared graphically.

### 3.1. Static Bedding Modulus

For all tested UBM samples the values of static bedding modulus were determined (Table 8). The following assumptions were made:

- DIN [23]: applied load range 0–0.25 N/mm^2^, assessed load ranges 0.02–0.10 N/mm^2^ (for *C*_stat_) and 0.02–0.20 N/mm^2^ (for *C*_tend_);
- EN [24]: applied load range 0.02–0.25 N/mm^2^, assessed load ranges 0.02–0.10 N/mm^2^ (for *C*_stat_) and 0.02–0.20 N/mm^2^ (for *C*_tend_).

**Figure 3:**
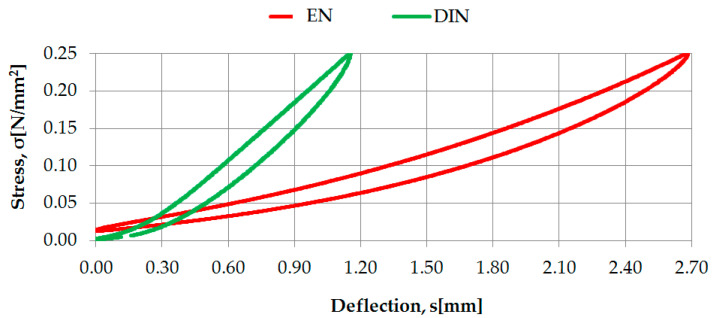
Static characteristics of UBM 042 determined according to DIN [23] and EN [24].

**Figure 4:**
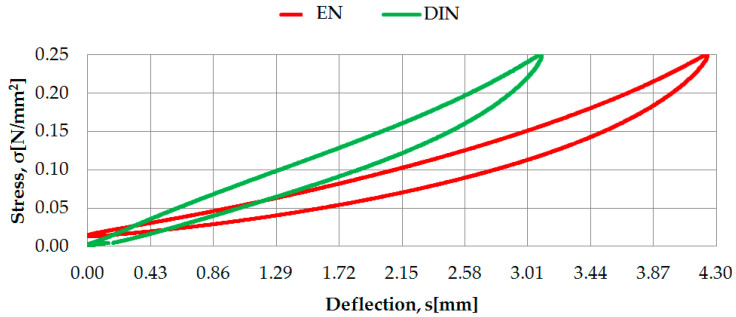
Static characteristics of UBM 045 determined according to DIN [23] and EN [24].

Examples of the identification of static characteristics of two selected rubber-based UBM samples (042 and 045), according to two considered regulations, are presented in Figure 3 and Figure 4.

### 3.2. Dynamic Bedding Modulus in Low Frequencies

For all tested UBM samples the values of dynamic bedding modulus in low frequencies were determined (Table 9). A dynamic bedding modulus $C_{\mathrm{dyn}}\;\left[N/{\mathrm{mm}}^{3}\right]$ is a ratio of the dynamic load with a specified value and frequency, applied to the sample with a certain cross-sectional area, to the sample deflection caused by this load. It characterizes deflection of the track system under a moving rolling stock. The following assumptions were made in both testing procedures: applied and assessed load range 0.02–0.10 N/mm^2^.

**Figure 5:**
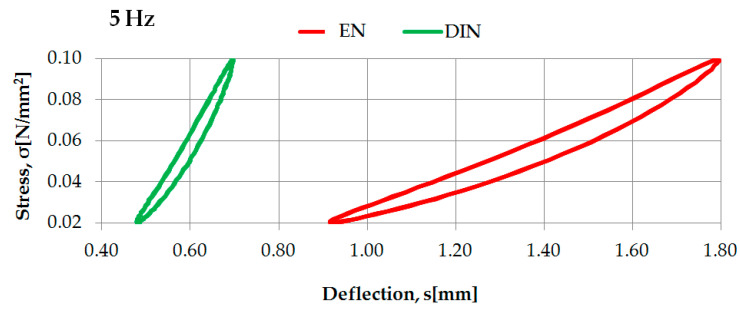
Dynamic characteristics in low frequencies of UBM 042 determined according to DIN [23] and EN [24].

**Figure 6:**
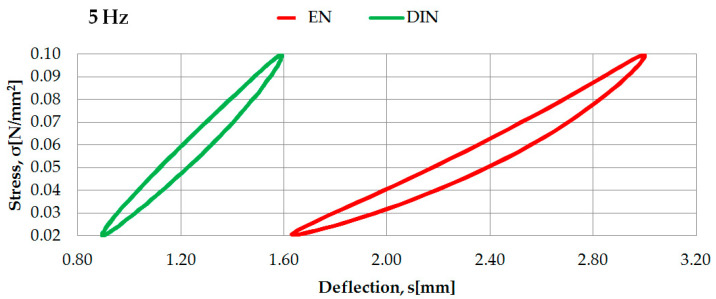
Dynamic characteristics in low frequencies of UBM 045 determined according to DIN [23] and EN [24].

Examples of the identification of dynamic characteristics in low frequencies of two selected rubber-based UBM samples (042 and 045), according to two considered regulations, are presented in Figure 5 and Figure 6.

### 3.3. Dynamic Bedding Modulus in High Frequencies

For all tested UBM samples the values of dynamic bedding modulus in high frequencies were determined (Table 10). The following assumptions were made in both testing procedures: initial static load *σ*_pre_ = 0.10 N/mm^2^ and constant velocity of vibration 100 dB compared to the reference value 5 × 10^−8^ m/s according to EN ISO 10846-2 [37].

**Figure 7:**
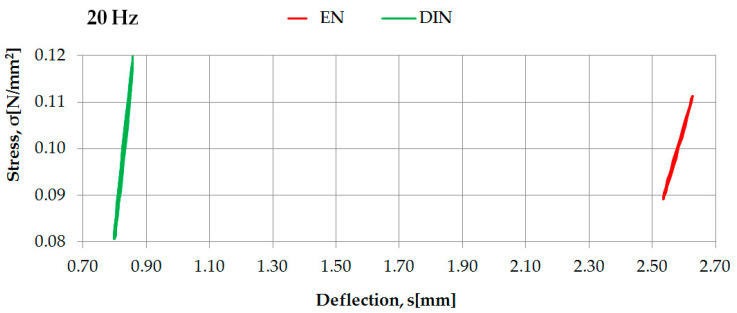
Dynamic characteristics in high frequencies of UBM 042 determined according to DIN [23] and EN [24].

**Figure 8:**
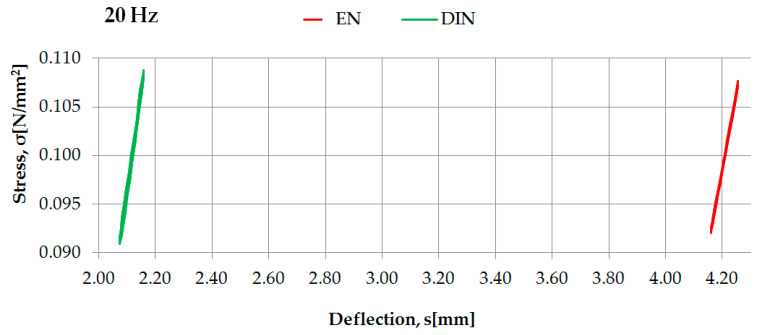
Dynamic characteristics in high frequencies of UBM 045 determined according to DIN [23] and EN [24].

Examples of the identification of dynamic characteristics in the high frequencies of two selected rubber-based UBM samples (042 and 045), according to two considered regulations, are presented in Figure 7 and Figure 8.

### 3.4. Discussion of Results

The performed laboratory tests, aimed at identifying discrepancies between two testing procedures: DIN [23] and EN [24], allowed the authors to formulate the following conclusions:

- The results differ because of two different ballast plates applied in both testing procedures—a flat plate (DIN) and GBP (EN); the use of GBP leads to more reliable results as this plate simulates the conditions under the ballast layer, but it requires creating new requirements by the railway infrastructure managers;
- The same load applied concentrically to the UBM sample (300 mm × 300 mm) with GBP causes much bigger deflections than in the case of the flat plate;
- Big differences in the values of static and dynamic (in low frequencies) bedding moduli determined using two approaches (from over 30% up to ~75%) were observed in the case of very stiff and medium types of UBM (Table 8 and Table 9); the identification of dynamic bedding moduli in high frequencies revealed smaller discrepancies of results—for the medium type below 25% (Table 10);
- The fact that smaller discrepancies of the values C_H_ than C_dyn_ were obtained, is a positive effect because it results in smaller influence on the analysis of vibration isolation effectiveness—the values that are used in the rheological model are C_H_, not C_dyn_;
- Medium differences in the values of static and dynamic bedding moduli (from ~10% up to ~30%) were observed in the case of soft types of UBM (Table 8, Table 9 and Table 10);
- Small differences in the values of static and dynamic bedding moduli (from 0% up to ~20%) were observed in the case of very soft types of UBM (Table 8, Table 9 and Table 10).;
- Small discrepancies of bedding moduli obtained for softer mats is a positive effect, as these mats (soft and medium types) are dedicated to be used as vibration isolators and for these mats the insertion loss (IL) should be determined; in the case of stiffer mats, for which the differences of the bedding moduli are bigger, there is no need to analyze IL, as they are dedicated for protection of the ballast layer;
- The obtained divergence of the parameters, determined using two testing procedures: DIN [23] and EN [24], confirms the need of retesting the mats that were previously tested in accordance to DIN [23] or UNI [27,28] using the new standard EN [24], as these parameters cannot be directly compared;
- The results obtained from the EN [24] procedure are more reliable than the ones determined according to DIN [23], as the geometric ballast plate (GBP) more accurately simulates the conditions in which UBM work;
- The protective function of UBM (protection against track degradation) dominates over the isolating function (vibration isolation) when the mat is stiff enough—it should have a high bedding modulus; according to Table 1 mats no. 42 and 45 tested according to DIN could be applied as the protection of the ballast, however if they were tested according to EN, they would be too soft for this function (see values in Table 8).

## 4. Mechanical Model of the Structure

**Figure 9:**
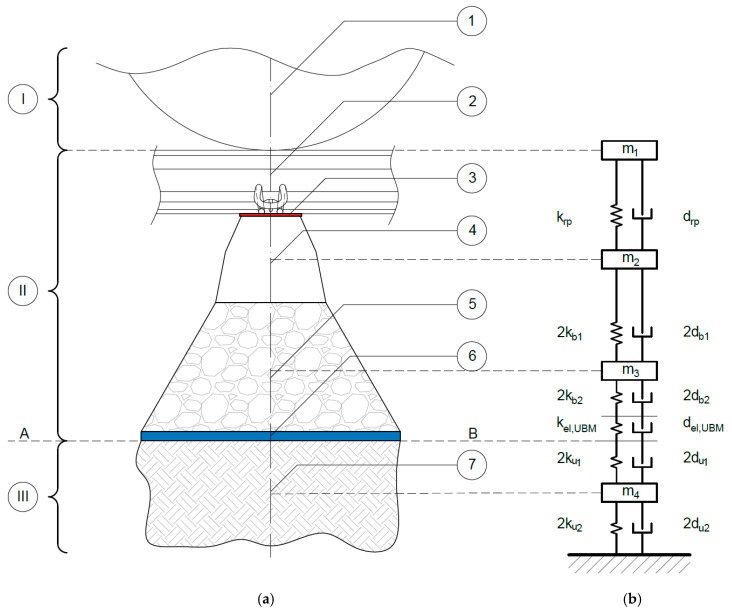
Ballasted track structure with UBM: (**a**) scheme of the structure; (**b**) mechanical model. Symbols: A-B—subgrade surface layer; I—rolling stock, II—railway track structure, III—subgrade; 1—non-spring part of the rolling stock, 2—rail, 3—rail pad, 4—sleeper, 5—ballast, 6—UBM, 7—subgrade.

Material characteristics identified in laboratory tests (see Section 3) were used to develop a rheological model of the considered vibration isolator—UBM—with the use of fractional elements and the analytical model of the ballasted track structure. The model was aimed at determining the insertion loss factor according to the works [2,3,4,38] and the standard DIN V 45673-4 [39]. However, in the present paper the authors propose a model that is much more advanced and gives more accurate results than the one described in [39]. The model (Figure 9) has four degrees of freedom (4-DoF) corresponding to four material points with the following masses: $m_{1}$—rail and rolling stock, $m_{2}$—sleeper, $m_{3}$—ballast, $m_{4}$—soil subgrade. The elastic and dissipative elements, modeled with rheological Kelvin-Voigt (KV) systems, characterize the properties of: rail pad ($k_{\mathrm{rp}}$ and $d_{\mathrm{rp}}$), ballast ($k_{b}$ and $d_{b}$), UBM ($k_{\mathrm{el},\mathrm{UBM}}$ and $d_{\mathrm{el},\mathrm{UBM}}$) and subsoil ($k_{u}$ and $d_{u}$).

In this section results of simulations performed using the novel rheological model are presented. The calculations were carried out in the program developed by the authors in Matlab environment (Matlab 2018b). The main aim of the simulations was to identify the influence of the testing procedure of static and dynamic elastic characteristics of under ballast mats on the level of vibration suppression—the insertion loss factor.

The presented results are limited to two selected UBM samples (042 and 045), which reveal the biggest discrepancies in the values of static and dynamic parameters determined using two considered approaches (DIN and EN)—Table 8 and Table 10.

**Figure 10:**
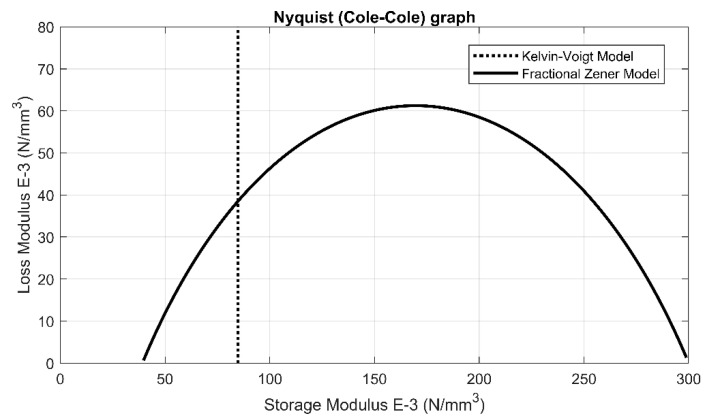
Complex plane characteristics of UBM modeled with Kelvin–Voigt and fractional zener model.

**Figure 11:**
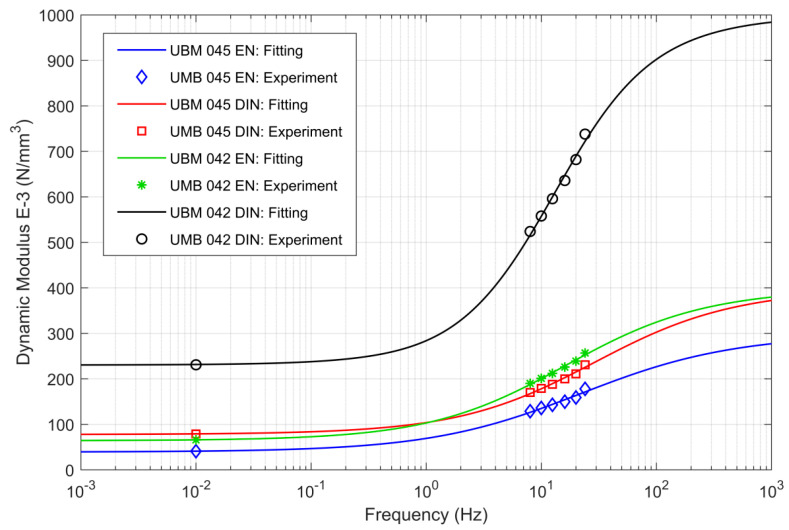
Curve fitting results for two analyzed UBM (042 and 045) models using two different procedures DIN [23] and EN [24].

In Figure 10 results of the curve fitting procedure, performed using laboratory tests results, are presented. This procedure is necessary to obtain rheological parameters of the analyzed UBM and has to be performed before the analysis of dynamic characteristics of vibration isolators using the mechanical model. The curve fitting procedure was performed using the Fractional Zener Model of UBM, similar to the fractional model of USP proposed in [40]. A simplified KV model that is presented in Figure 9 is not suitable here, as it can be tuned for just one frequency or a mean of the results obtained for several frequencies can be taken. It can be proved that the KV model characteristics in complex plain (Cole–Cole or Nyquist graph) are unrealistic. As we can see in Figure 10 the storage modulus value is constant (does not depend on frequency of excitation). This is not suitable for realistic behavior of visco-elastic materials [41]. On the other hand, in case of any UBM model fitted by using the fractional zener model, the characteristics visualized in Figure 11 are reasonable and depend on excitation frequencies.

The fractional zener model includes a rheological element defined by fractional derivatives. Constitutive relationships of this fractional element (spring-pot), relating the force f(t) and the displacement $u_{\mathrm{sp}}\left(t\right)$, may be expressed in differential form:

(1) $$f\left(t\right):=d_{\mathrm{sp}}D^{\alpha }u_{\mathrm{sp}}\left(t\right);\;\alpha \in \left(0,1\right),$$

where α and $d_{sp}$ are fractional element parameters, while $D^{\alpha }\equiv \frac{d^{\alpha }}{dt^{\alpha }}$ denotes fractional derivative operator defined as follows:

(2) $$D^{\alpha }u_{sp}\left(t\right):=\frac{u_{sp}\left(0\right)}{\Gamma \left(1-\alpha \right)\;t^{\alpha }}+\frac{1}{\Gamma \left(1-\alpha \right)}\;\int_{0}^{t}\frac{{\dot{u}}_{sp}\left(\tau \right)}{{\left(t-\tau \right)}^{\alpha }}\;d\tau$$

(3) $$\Gamma \left(1-\alpha \right):=\int_{0}^{\infty }t^{-\alpha }e^{-t}dt.$$

Putting α=1 leads to a classical dashpot model, while for α=0 a simple spring element is obtained. The use of the fractional model of UBM makes it possible to conduct the curve fitting procedure for a series of frequencies, as presented in Figure 11.

**Figure 12:**
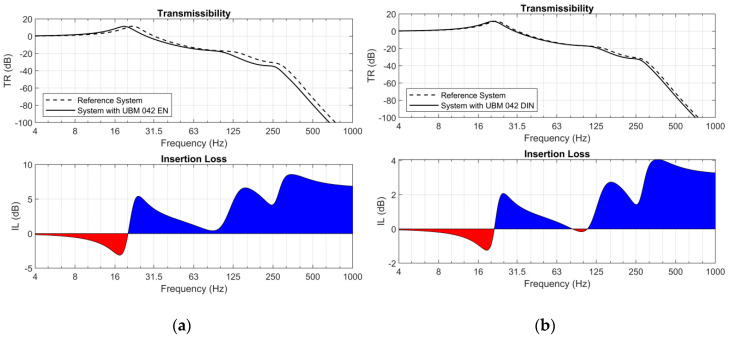
Transmissibility (TR) and insertion loss (IL)—reference system and system with UBM 042: (**a**) according to EN [24]; (**b**) according to DIN [23].

**Figure 13:**
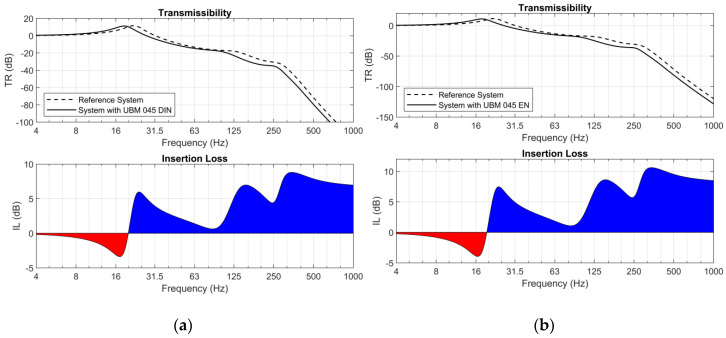
Transmissibility (TR) and insertion loss (IL)—reference system and system with UBM 045: (**a**) according to EN [24]; (**b**) according to DIN [23].

Moreover, for each analyzed sample two diagrams were determined (Figure 12 and Figure 13): transmissibility (TR) and insertions loss (IL) functions [38]. In both cases two approaches were used: the procedures described in DIN 45673-5 [23] and EN 17282 [24]. The insertion loss diagrams indicate the regions of effective vibration isolation and regions where the application of the analyzed mats can deteriorate the vibration isolation capacity in the systems (negative values of insertion loss marked in red color). Similar analyses were carried out for USP and presented in [40].

The values of insertion loss (IL) were analyzed in two ranges of frequencies:

- 31.5–63 Hz—vibration;
- 63–125 Hz—secondary structure-borne noise.

The results of the analysis of vibration suppression are presented in Table 11.

It should be noticed that regardless of the approach, there are no significant differences between the frequencies ranges for which insertion loss has positive values. The transition frequencies obtained using DIN and EN approaches differ by less than 1 Hz. However, choice of the approach affects the effectiveness of vibration suppression understood as the values of insertion loss.

Analysis of the mechanical model of the structure indicated that the testing procedure has a significantly bigger influence on the results in the case of stiffer mats. The softer the element, the smaller the discrepancies of the obtained values are. However, the observed phenomenon should be considered as positive. Softer mats are used mainly as vibration isolators and for these mats the analysis of IL is crucial. Stiffer mats, on the other hand, are aimed mainly at protection of the ballast layer, and there is no need to assess the vibration isolation effectiveness in their case.

## 5. Conclusions

The present paper was aimed at determining static and dynamic characteristics of prototype UBM using two approaches: a procedure described in the commonly used German standard DIN 45673-5 [23] and the one included in the new European standard EN 17282 [24], which was released in October 2020.

First, laboratory tests of eighteen UBM samples were presented. The tests were performed in order to find static and dynamic (in low and high frequencies) bedding moduli and to indicate discrepancies of the results obtained according to the considered procedures DIN and EN. Major discrepancies (from over 30% up to ~75%) in the values of static and dynamic characteristics were demonstrated in the case of mats with higher stiffness (medium, stiff and very stiff types). The softer the tested element, the smaller differences between the obtained results were observed: at the level of ~10% up to ~30% for soft mats and 0% to ~20% for very soft mats.

Apart from the laboratory tests results, an original approach to the analysis of ballasted track systems with UBM was proposed as a method for assessing the level of vibration suppression of the tested UBM. It is a novel 4-DoF mechanical model of the system that includes a fractional rheological model of UBM. Usually, railway track structures with vibration isolators are analyzed using discrete mechanical models with few degrees of freedom. Here the authors present an analytical model that has 4-DoF which correspond to four material points: rail and rolling stock, sleeper, ballast and soil subgrade. Rheological Kelvin–Voigt systems are used to model the elastic and dissipative elements. Additionally, the model contains components that represent the UBM. The proposed model simulates with great precision behavior of the structure and is much more accurate than typical models described in the literature.

The main aim of the simulations was to identify the influence of the testing procedure of static and dynamic elastic characteristics of UBM on the level of vibration suppression—the insertion loss factor. For two selected UBM samples, a very stiff type and a medium type, dynamic characteristics such as transmissibility and insertions loss functions were determined. Moreover, the values of insertion loss for the frequencies in the 1/3 octave bands were given, which allowed the authors to assess the discrepancies between the two testing procedures. Similar to the laboratory tests results, the analysis of the isolated structure using the developed mechanical model indicated that the choice of the testing procedure is crucial in the case of stiffer mats. For soft and very soft mats, smaller discrepancies of the results were obtained.

## Figures and Tables

**Table 1:** Requirements for static bedding moduli of under ballast mats (UBM) used as protection of the ballast layer, according to DB regulations [25,26].

*V*_max_ [km/h]	Static Bedding Modulus *C*_stat_ (N/mm^3^)
>230	<0.10; 0.15>
≤230	<0.10; 0.25>

**Table 2:** Requirements for static bedding moduli of UBM used as vibration isolators, according to DB regulations [1,33,34].

*V*_max_ [km/h]	Axle Load [kN]	Static Bedding Modulus *C*_stat_ [N/mm^3^]
≤120	≤160	0.02
≤120	>160 *	0.03
(120; 200)		0.06
≥200		0.10

* Regulations of DB Netz TM 2010-1564 I.NVT 4 [34] specify ranges of axle loads below 16 t and below 25 t.

**Table 3:** Requirements for static bedding moduli of UBM according to ÖBB regulations [35].

*V*_max_ [km/h]	Line Type	Static Bedding Modulus *C*_stat_ [N/mm^3^]
≤120	Main lines	≥0.025
(120; 200>)		≥0.060
>200		≥0.100

**Table 4:** Requirements for static bedding moduli of UBM according to SBB CFF FFS [1,36].

*V*_max_ [km/h]	Function	Static Bedding Modulus *C*_stat_ [N/mm^3^]
≤120	Vibration isolation	≥0.03
(120; 200)		≥0.06
≥200		≥0.10

**Table 5:** Coloristic identification of UBM types based on the values of static bedding modulus, according to DIN 45673-5 [23].

	Very soft for 0 < *C*_stat_ < 0.03 N/mm^3^
	Soft for 0.03 ≤ *C*_stat_ < 0.06 N/mm^3^; for *V*_max_ ≤ 120 km/h
	Medium for 0.06 ≤ *C*_stat_ < 0.10 N/mm^3^, for 120 km/h < *V*_max_ < 200 km/h
	Stiff for 0.10 ≤ *C*_stat_ < 0.15 N/mm^3^, for *V*_max_ ≥ 200 km/h
	Very stiff for 0.15 ≤ *C*_stat_ < 0.25 N/mm^3^

**Table 6:** Properties of tested UBM samples.

UBM Sample No.	UBM Material	Density [kg/m^3^]	Thickness [mm]
37	mineral wool	230	35
38	mineral wool	230	35
39	mineral wool	230	35
42	SBR	850	25
43	SBR	650	25
44	SBR	700	15
45	SBR	700	20
46	SBR	700	25
47	SBR	700	30
48	SBR	600	15
49	SBR	600	20
50	SBR	600	25
51	SBR	600	30
52	SBR	500	15
53	SBR	500	20
54	SBR	500	25
55	SBR	500	30
56	SBR	550	20

**Table 7:** Coloristic classification of UBM based on the variations of bedding moduli determined according to different testing procedures.

	Δ*C* < 25%
	25% ≤ Δ*C* < 50%
	50% ≤ Δ*C* < 75%
	Δ*C* ≥ 75%

**Table 8:** Static bedding moduli of UBM according to DIN [23] and EN [24].

UBM Sample No.	UBM Type	C_stat_ DIN [N/mm^3^]	C_tend_ DIN [N/mm^3^]	C_stat_ EN [N/mm^3^]	C_tend_ EN [N/mm^3^]	ΔC_stat_ [%]	ΔC_tend_ [%]
37	Very soft	0.028	0.043	0.027	0.041	−3.6	−4.7
38	Very soft	0.026	0.037	0.028	0.040	7.7	8.1
39	Very soft	0.023	0.034	0.026	0.038	13.0	11.8
42	Very stiff	0.231	0.256	0.066	0.083	−71.4	−67.6
43	Medium	0.072	0.072	0.037	0.045	−48.6	−37.5
44	Very stiff	0.158	0.158	0.056	0.074	−64.6	−53.2
45	Medium	0.079	0.080	0.041	0.051	−48.1	−36.3
46	Medium	0.075	0.073	0.039	0.047	−48.0	−35.6
47	Medium	0.065	0.062	0.037	0.043	−43.1	−30.6
48	Soft	0.056	0.067	0.040	0.053	−28.6	−20.9
49	Soft	0.037	0.046	0.029	0.039	−21.6	−15.2
50	Soft	0.037	0.043	0.028	0.036	−24.3	−16.3
51	Soft	0.03	0.035	0.024	0.030	−20.0	−14.3
52	Soft	0.032	0.047	0.032	0.046	0.0	−2.1
53	Very soft	0.025	0.036	0.025	0.036	0.0	0.0
54	Very soft	0.02	0.029	0.020	0.029	0.0	0.0
55	Very soft	0.016	0.023	0.016	0.023	0.0	0.0
56	Soft	0.031	0.041	0.025	0.035	−19.4	−14.6

**Table 9:** Dynamic bedding moduli in low frequencies of UBM according to DIN [23] and EN [24].

No.	C_dyn_ DIN [N/mm^3^]			C_dyn_ EN [N/mm^3^]			ΔC_dyn_ [%]		
	5 Hz	10 Hz	20 Hz	5 Hz	10 Hz	20 Hz	5 Hz	10 Hz	20 Hz
37	0.038	0.040	0.051	0.033	0.034	0.039	−13.2	−15.0	−23.5
38	0.032	0.034	0.044	0.034	0.036	0.042	6.3	5.9	−4.5
39	0.030	0.031	0.046	0.032	0.033	0.040	6.7	6.5	−13.0
42	0.383	0.406	0.439	0.093	0.097	0.111	−75.7	−76.1	−74.7
43	0.114	0.121	0.134	0.055	0.058	0.071	−51.8	−52.1	−47.0
44	0.258	0.272	0.298	0.081	0.085	0.099	−68.6	−68.8	−66.8
45	0.123	0.130	0.143	0.059	0.062	0.076	−52.0	−52.3	−46.9
46	0.116	0.123	0.136	0.055	0.057	0.070	−52.6	−53.7	−48.5
47	0.100	0.105	0.117	0.054	0.057	0.068	−46.0	−45.7	−41.9
48	0.089	0.094	0.107	0.061	0.064	0.078	−31.5	−31.9	−27.1
49	0.059	0.062	0.073	0.044	0.046	0.057	−25.4	−25.8	−21.9
50	0.059	0.062	0.073	0.042	0.045	0.056	−28.8	−27.4	−23.3
51	0.048	0.050	0.060	0.036	0.038	0.046	−25.0	−24.0	−23.3
52	0.058	0.061	0.074	0.051	0.054	0.066	−12.1	−11.5	−10.8
53	0.045	0.048	0.059	0.040	0.043	0.055	−11.1	−10.4	−6.8
54	0.036	0.038	0.048	0.033	0.035	0.046	−8.3	−7.9	−4.2
55	0.028	0.030	0.047	0.027	0.029	0.038	−3.6	−3.3	−19.1
56	0.054	0.057	0.069	0.043	0.045	0.057	−20.4	−21.1	−17.4

**Table 10:** Dynamic bedding moduli in high frequencies of UBM according to DIN [23] and EN [24].

No.	C_H_ DIN [N/mm^3^]			C_H_ EN [N/mm^3^]			ΔC_H_ [%]		
	12.5 Hz	16 Hz	20 Hz	12.5 Hz	16 Hz	20 Hz	12.5 Hz	16 Hz	20 Hz
37	0.169	0.178	0.194	0.152	0.162	0.168	−10.1	−9.0	−13.4
38	0.113	0.119	0.125	0.100	0.104	0.111	−11.5	−12.6	−11.2
39	0.123	0.129	0.136	0.118	0.124	0.130	−4.1	−3.9	−4.4
42	0.596	0.636	0.682	0.212	0.226	0.239	−64.4	−64.5	−65.0
43	0.176	0.185	0.195	0.134	0.141	0.149	−23.9	−23.8	−23.6
44	0.360	0.383	0.407	0.208	0.220	0.236	−42.2	−42.6	−42.0
45	0.188	0.200	0.211	0.143	0.150	0.159	−23.9	−25.0	−24.6
46	0.175	0.184	0.195	0.133	0.140	0.147	−24.0	−23.9	−24.6
47	0.147	0.155	0.164	0.117	0.123	0.131	−20.4	−20.6	−20.1
48	0.203	0.218	0.233	0.179	0.191	0.204	−11.8	−12.4	−12.4
49	0.146	0.155	0.165	0.127	0.135	0.143	−13.0	−12.9	−13.3
50	0.128	0.135	0.144	0.114	0.121	0.128	−10.9	−10.4	−11.1
51	0.103	0.108	0.116	0.094	0.100	0.107	−8.7	−7.4	−7.8
52	0.203	0.221	0.235	0.182	0.194	0.208	−10.3	−12.2	−11.5
53	0.172	0.183	0.195	0.147	0.155	0.165	−14.5	−15.3	−15.4
54	0.133	0.141	0.151	0.102	0.109	0.119	−23.3	−22.7	−21.2
55	0.109	0.116	0.125	0.102	0.108	0.115	−6.4	−6.9	−8.0
56	0.165	0.174	0.187	0.148	0.158	0.169	−10.3	−9.2	−9.6

**Table 11:** Suppression of the vibration level: insertion loss values obtained for two UBM samples from the 4-DoF mechanical model in 1/3 octave bands.

UBM Type	Frequencies in 1/3 Octave Bands *f* [Hz]							
	31.5 Hz		63 Hz		125 Hz		IL > 0 (DIN) [Hz]	IL > 0 (EN) [Hz]
	IL (DIN) [dB]	IL (EN) [dB]	IL (DIN) [dB]	IL (EN) [dB]	IL (DIN) [dB]	IL (EN) [dB]		
very stiff	1.4	3.6	0.4	1.2	1.2	4.7	21.1	20.1
medium	4.0	4.9	1.4	1.8	5.1	6.8	19.9	19.3

## Data Availability

The data presented in this study are available on request from the corresponding author. These are results of laboratory tests carried out by the authors within the project mentioned above.
